# *Southern African Journal of Infectious Diseases* – Yesterday, today and tomorrow

**DOI:** 10.4102/sajid.v34i1.182

**Published:** 2019-12-12

**Authors:** Charles Feldman

**Affiliations:** 1 Department of Internal Medicine, Faculty of Health Sciences, University of the Witwatersrand, Johannesburg, South Africa

There have been a number of editorials written over the years for the *Southern African Journal of Infectious Diseases* by the editors, mostly to describe changes that have been made to the journal. These have also been taken as an opportunity to chronicle the history of the journal so that we do not forget where the journal has come from and appreciate where we are headed in the future. This editorial will not deviate much from that pattern. The title of one of the previous editorials was ‘The journal – past, present, future’; therefore, rather than using the same title, I ruminated on whether I should call it ‘the good, the bad and the ugly’, but eventually settled on ‘yesterday, today and tomorrow’.

A number of changes have occurred in the journal since I took over in 2003 as the editor-in-chief. These ‘yesterday’ events included making changes to the name and look of the journal, changes to the editorial board and bringing the journal into the world of ‘open access’. The latter is not only the way that most journals are going, but is also an important consideration as many of the big funders of research require study findings to be published immediately in open access journals. It is easy to understand the reason why much of this research funding comes from taxpayers’ money and therefore the funders believe, quite rightly, that the study outcomes should be placed in the public domain as soon as possible after study completion.

One of the most significant ‘today’ events is that Prof. Hendrik Koornhof elected to retire as the emeritus editor of the journal after a very long association with the journal, including a stint of approximately 15 years as editor-in-chief of the journal. The total contribution that Prof. Koornhof has made, not only to the journal but also to infectious diseases in South Africa, is enormous and cannot be adequately expressed in words that would do it justice. We are all indebted to him and owe him our gratitude.

Other changes listed as ‘today’ events in this editorial include the fact that we have changed publishers. We have moved from Medpharm Publications to AOSIS. The reasons for this are purely financial and logistic, rather than any concerns about expertise, professionalism or interpersonal relationships. The editorial committee have enjoyed being with, and interacting with, Medpharm Publications and their staff over all these years. The journal owes a considerable ‘thank you’ to Medpharm Publications, which carried our journal for many years, despite tough economic times, and kept us afloat.

It is a harsh reality that the greatest challenge facing journals worldwide is financial viability. No longer can journals rely on the income generated from advertising revenue. This is particularly true in the field of infectious diseases, a speciality in which one of the most commonly prescribed agents is an antibiotic, many of which are genericised, and not terribly expensive. This has impacted significantly on our ability to generate income through advertising. It is for this reason that the other bit of ‘today’ changes we have made is to start charging ‘page fees’/‘article processing charges’ for manuscripts submitted to, and accepted, in the journal from the beginning of 2019. Fortunately many of the universities in South Africa, and elsewhere, have set up measures for supporting open access publishing and many of the funders also allow applicants to build publication costs into their funding applications. It is pleasing to see that despite the introduction of page charges, new submissions to our journal do not seem to be tailoring off. The rate of articles submitted from sub-Saharan Africa, and even outside Africa is also still on the increase.

The most important ‘tomorrow’ issue that we need to concentrate on is getting the journal indexed in PubMed. Fortunately, AOSIS is highly experienced in doing this and has taken a number of journals through the indexing process. This is our most immediate and urgent action in the near future.

